# Incidence and risk factors of perioperative deep vein thrombosis in patients undergoing primary hip arthroplasty via the direct anterior approach

**DOI:** 10.1186/s13018-023-04443-8

**Published:** 2024-01-03

**Authors:** Zaikai Zhuang, Qiangqiang Li, Yao Yao, Ying Shen, Dongyang Chen, Qing Jiang

**Affiliations:** 1 Division of Sports Medicine and Adult Reconstructive Surgery, Department of Orthopedic Surgery, Nanjing Drum Tower Hospital Clinical College of Nanjing Medical University, 321 Zhongshan Road, Nanjing, 210008 Jiangsu People’s Republic of China; 2 State Key Laboratory of Pharmaceutical Biotechnology, Nanjing University, Nanjing, Jiangsu People’s Republic of China; 3 Branch of National Clinical Research Center for Orthopedics, Sports Medicine and Rehabilitation, Nanjing, People’s Republic of China

**Keywords:** Deep vein thrombosis, Hip arthroplasty, Direct anterior approach, Risk factors

## Abstract

**Background:**

Deep vein thrombosis (DVT) is a frequent complication following hip arthroplasty. There still has been a lack of studies analyzing the perioperative risk factors of DVT following hip arthroplasty via direct anterior approach (DAA).

**Methods:**

Patients who underwent unilateral primary hip arthroplasty via DAA in our hospital from August 2015 to January 2022 were included. Patients’ data, including demographic data, clinical features, past medical history, operative data, and laboratory data, were analyzed and compared between patients with and without DVT. Logistic regression analysis was conducted to identify the independent risk factors. Receiver operating characteristic (ROC) curve analysis was used to assess the best cutoff value of continuous variables with statistical significance.

**Result:**

A total of 651 patients were included. The incidence of DVT before and after hip arthroplasty was 12.7% and 6.7%, respectively. Logistic regression analysis indicated that age ≥ 65 years (OR 4.594, 95% CI 1.994–10.587), women (OR 2.331, 95% CI 1.285–4.227), and cerebral infarction (OR 1.984, 95% CI 1.138–3.460) were independent risk factors for preoperative DVT. And age ≥ 65 years (OR 4.859, 95% CI 1.062–22.226), tumor (OR 3.622, 95% CI 1.108–11.841), and preoperative *D*-dimer (OR 1.040, 95% CI 1.004–1.078) were risk factors for postoperative DVT. The ROC curve analysis showed that the best cutoff value of preoperative *D*-dimer for the diagnosis of postoperative DVT is 1.44 mg/L.

**Conclusions:**

The incidence of DVT in patients undergoing DAA hip arthroplasty was low and the occurrence of DVT before and after unilateral primary hip arthroplasty performed through DAA was related to multiple factors.

## Introduction

Hip arthroplasty is a common surgical procedure in orthopedic surgery to treat hip diseases [1, 2]. As the population ages and the prevalence of the above hip diseases are rising quickly, a large number of people have to receive hip arthroplasty treatment [3]. More than 1,000,000 total hip arthroplasties (THAs) are performed annually worldwide [4]. With more patients now undergoing joint arthroplasty, a postoperative complication such as deep vein thrombosis (DVT) has also increased accordingly. The direct anterior approach (DAA) is a more minimally invasive operative approach for hip arthroplasty and is considered to be the least traumatic to the muscle tissues with less postoperative pain, faster recovery, and shorter hospitalization [5–7]. Theoretically, the incidence of DVT is expected to decrease accordingly. Previous studies have only focused on the incidence and risk factors for postoperative DVT after hip arthroplasty via conventional or other unknown approaches [8, 9]. So far, there still has been a lack of studies analyzing the risk factors for DVT after hip arthroplasty via DAA.

It has been reported that the incidence of DVT in patients following hip arthroplasty without thromboprophylaxis is approximately 42–57% [10]. DVT can cause long-term complications such as post-thrombotic syndrome [11–13] and fatal complications such as pulmonary embolism [14], the latter of which not only brings negative consequences to the patients but also generates a heavy economic burden to other family members and the healthcare system [15, 16]. Therefore, identifying the risk factors and early intervention of DVT emerge as an important method to avoid further damage. However, most previous studies have only focused on the analysis of risk factors and prevention of DVT before or after hip arthroplasty separately. Relatively fewer studies focused on the analysis of risk factors throughout the entire perioperative period, possibly leading high-risk patients to miss effective intervention before surgery.

Therefore, we retrospectively reviewed perioperative data of patients who underwent primary hip arthroplasty via DAA in our hospital to analyze their clinical characteristics and explore the preoperative and postoperative DVT risk factors. We aimed to provide a reference for the early identification of risk factors and individualized thromboprophylaxis to further reduce the perioperative incidence of DVT in patients undergoing hip arthroplasty.

## Methods

### Ethics

All methods were carried out in accordance with the Declaration of Helsinki. Informed consent to participate was obtained from all patients included in the study. All procedures were performed by the same senior surgeon with extensive experience in hip arthroplasty. This study had been approved by the Medical Ethics Committee of our hospital.

### Patients

**Fig. 1:**
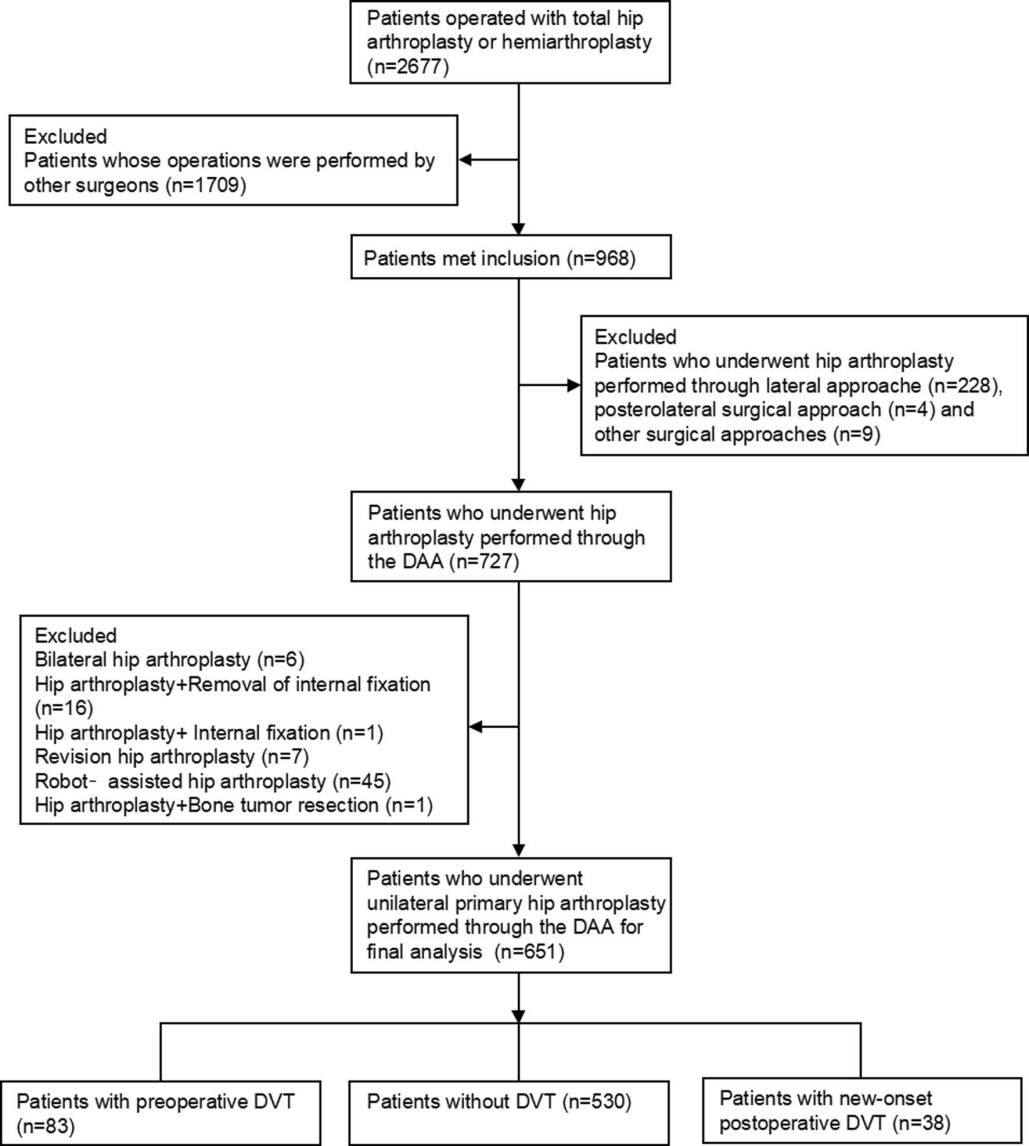
The detailed approach for participants’ selection

From August 2015 to January 2022, a total of 2677 patients underwent hip arthroplasty in our joint center. We excluded 1709 patients who had hip arthroplasty by other surgeons; 241 patients who underwent hip arthroplasty performed through other surgical approaches; 76 patients who had other surgical modalities, leaving 651 patients who underwent unilateral primary hip arthroplasty performed through DAA for analyses. The detailed participants’ selection process is shown in Fig. 1.

### The diagnosis of DVT of the lower extremity

Once admission, a Sonosite M-Turbo ultrasound system was used to perform Doppler ultrasound examinations on the lower limbs in all patients. An ultrasound scan was performed 1 day preoperatively, on the first and third postoperative day and on the day of discharge from hospital. Loss of vein compressibility was considered diagnostic evidence of DVT. Routine scanning was performed for the proximal vein and distal vein thrombosis of bilateral lower extremities.

### DVT preventions

Of these patients who met the inclusion criteria, 52 patients were taking antiplatelet or anticoagulant medications preoperatively. All these medications were discontinued and changed to the low molecular weight heparin (0.4 ml/d) upon admission. Eight hours after operation, all patients were given low molecular weight heparin (0.4 ml/d) daily until the day of discharge (For patients who developed subcutaneous hematoma or patients with poor drug compliance, oral rivaroxaban (10 mg/d) daily was selected). All patients received pneumatic pump therapy applied to both lower extremity and performed ankle pumping exercises (200 times/day) postoperatively. From the first day after the surgery, patients were encouraged to start isometric contraction of muscles of the lower extremities and instructed to perform active and passive activities of joints. Drains were removed within 48 h after surgery, and patients were then encouraged to walk with a walking aid.

### Surgical procedures

All patients underwent hip arthroplasty by the same surgeon. Patients were placed in the supine position, and the operation was conducted under general anesthesia combined with a femoral nerve block. At operation, a line was drawn between the superolateral border of the patella and the anterior superior iliac spin on the same side. Then, an 8 to 10 cm incision was made at a level 3 cm parallelly outside this line. After incising the skin, the subcutaneous fat down to the deep fascia was incised, which is incised longitudinally. A blunt dissection was performed along the spaces between the tensor fascia lata and rectus femoris and then the branches of the lateral circumflex femoral artery were dissected and ligated. The fat surrounding the joint capsule was removed, and the muscle was carefully detached from the joint capsule to expose the joint capsule. Next, the joint capsule was opened to expose the femoral head and neck, an osteotomy of the femoral neck was done, and the femoral head was removed. For patients with total hip arthroplasty, the acetabulum was cleaned and then pared until blood oozing in the subchondral bone was visible, and the proper acetabular cup and acetabular liner were mounted. In further continuation, the affected limb was maintained by utilizing external rotation, adduction, and posterior extension position. After the proximal femur was lifted, the femoral components and femoral head prosthesis were implanted and reduced. Finally, the deep fascia, subcutaneous tissues, and skin were sutured layer by layer.

### Data collection

We collected patients’ information including age, gender, body mass index (BMI), history of hypertension, diabetes, cardiovascular disease, cerebral infarction, tumor and anticoagulation, surgical factors including duration of surgery, intraoperative blood loss, and postoperative information including postoperative bed rest time and postoperative drainage volume. The formation time, location, and type of DVT were also collected. The laboratory indicators collected before and after surgery included triglyceride level, cholesterol level, apolipoprotein A level, apolipoprotein B level, hemoglobin level, red blood cell (RBC) level, hematocrit (HCT) level, platelet level, activated partial thromboplastin time (APTT) level, prothrombin time (PT) level, thrombin time (TT) level, fibrinogen level, and *D*-dimer level. Of these, the postoperative *D*-dimer was measured on the third postoperative day, and other postoperative indicators were measured on the first day after surgery.

### Statistical analysis

Statistical analysis was carried out using SPSS v. 26 (IBM Corp., Armonk, NY, USA). Shapiro–Wilk test was used to determine whether the continuous data were normally distributed. Normally-distributed data were analyzed by independent-sample t-test, and the results were expressed as mean ± standard deviation (x ± s). Non-normally distributed data were tested by the Mann–Whitney *U* test, and the results were expressed as median (quartile) [M (Q1, Q3)]. Categorical variables were expressed as counts and evaluated using the Chi-squared test. The statistically significant variables in univariate analysis were then included in multivariate logistic regression analysis to determine independent risk factors. Then continuous variables with statistical significance in the multivariate logistic regression analysis were analyzed to assess the best cutoff value by ROC curves. *P* < 0.05 was the significance threshold for this study.

## Results

### The characteristics of included patients

A total of 651 patients undergoing unilateral primary hip arthroplasty through the DAA were finally included, 38.6% of whom were men. The mean age of the included patients was 68.3 years. And the average length from surgery to discharge and the overall hospital stay for the included patients were 4.8 and 8.5 days, respectively. The incidence of DVT before hip arthroplasty was 12.7%, and the occurrence of new-onset DVT after the surgery was 6.7%. All patients with DVT were asymptomatic. The distribution of perioperative DVT is summarized in Table 1.

**Table 1:** The location of perioperative DVT

	Pre-operation	Post-operation
Total, *n*	83	38
Affected proximal DVT, *n* (%)	3 (3.61%)	1 (2.63%)
Affected distal DVT, *n* (%)	47 (56.63%)	22 (57.89%)
Opposite proximal DVT, *n* (%)	3(3.61%)	1 (2.63%)
Opposite distal DVT, *n* (%)	15 (18.07%)	11 (28.95%)
Bilateral proximal DVT, *n* (%)	0	0
Bilateral distal DVT, *n* (%)	18 (21.69%)	3 (7.89%)

### The risk factors of preoperative DVT

The univariate analysis is shown in Table 2, and the results showed that there were significant differences in age ≥ 65 years (*P* < 0.001), women (*P* < 0.001), diabetes (*P* = 0.004), cardiovascular disease (*P* = 0.032), cerebral infarction (*P* < 0.001), triglyceride (*P* = 0.029), preoperative hemoglobin (*P* = 0.019), preoperative RBC (*P* = 0.046), preoperative HCT (*P* = 0.023), preoperative PT (*P* = 0.014), preoperative fibrinogen (*P* < 0.001), preoperative TT (P = 0.013), and preoperative *D*-dimer (*P* < 0.001) between DVT and non-DVT group.

**Table 2:** Univariate analysis of risk factors for preoperative DVT

Risk factors	DVT group (*n* = 83)	Non-DVT group (*n* = 568)	*P* Value
Age (≥ 65 years vs < 65 years)	76/7	335/233	< 0.001
Sex (women vs men)	66/17	334/234	< 0.001
BMI (≥ 25 vs < 25)	20/63	194/374	0.068
Hypertension (yes vs no)	42/41	229/339	0.076
Diabetes (yes vs no)	18/65	61/507	0.004
Cardiovascular disease (yes vs no)	17/66	68/500	0.032
Cerebral infarction (yes vs no)	28/55	89/479	< 0.001
Tumor (yes vs no)	3/80	26/542	0.691
Anticoagulation (yes vs no)	11/72	41/527	0.058
Triglyceride (mmol/L)	1.0(0.8, 1.2)	1.1(0.8, 1.5)	0.029
Cholesterol (mmol/L)	4.1(3.6, 4.9)	4.3(3.7, 5.0)	0.200
Apolipoprotein A (g/L)	1.0(0.9, 1.2)	1.0(0.9, 1.1)	0.965
Apolipoprotein B (g/L)	0.8(0.6, 0.8)	0.8(0.6, 0.9)	0.294
Preoperative hemoglobin (g/L)	120.0(112.5, 136.5)	128.0(117.0, 137.0)	0.019
Preoperative RBC (10^12/L)	4.0(3.7, 4.6)	4.2(3.9, 4.6)	0.046
Preoperative HCT (L/L)	36.5(33.5, 40.6)	38.3(35.1, 41.2)	0.023
Preoperative platelet (10^9/L)	183.0(144.5, 218.0)	190.0(155.5, 236.0)	0.232
Preoperative PT (sec)	11.6(11.1, 12.3)	11.3(10.8, 12.0)	0.014
Preoperative APTT (sec)	27.3(25.8, 30.3)	27.3(25.8, 29.1)	0.463
Preoperative fibrinogen (g/L)	4.0(3.4, 4.8)	3.2(2.7, 3.8)	< 0.001
Preoperative TT (sec)	17.3(16.3, 18.1)	17.6(16.9, 18.5)	0.013
Preoperative *D*-dimer (mg/L)	2.9(1.6, 5.7)	0.8(0.4, 2.0)	< 0.001

*BMI* Body mass index, *RBC* Red blood cell, *HCT* Hematocrit, *APTT* Activated partial thromboplastin time, *PT* Prothrombin time, *TT* Thrombin time

**Fig. 2:**
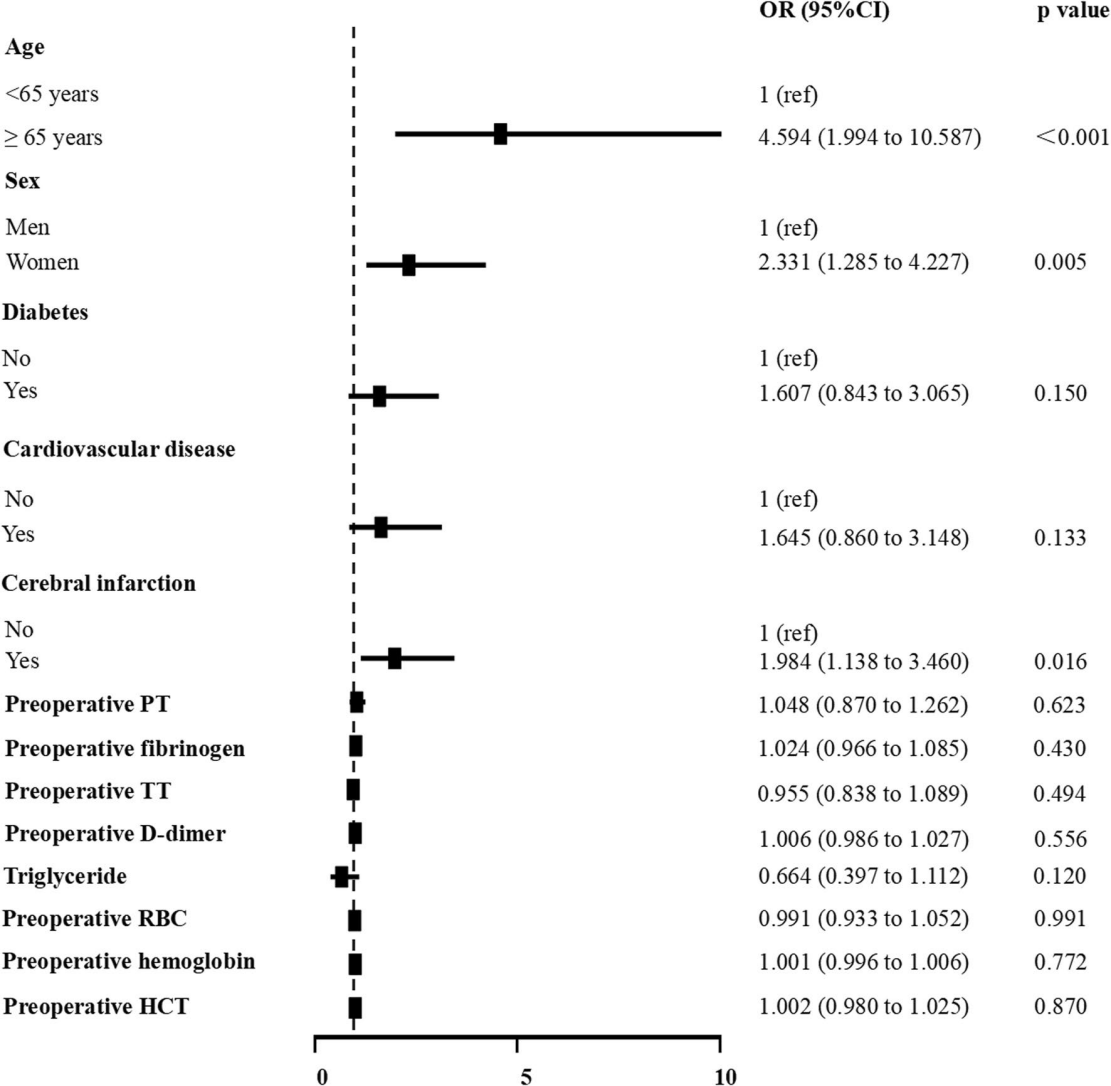
Multivariate analysis of preoperative DVT of lower limb in patients awaiting primary hip arthroplasty

We included these factors into further multivariate logistic regression analysis, and the results showed that age ≥ 65 years (OR 4.594, 95% CI 1.994–10.587), women (OR 2.331, 95% CI 1.285–4.227), and cerebral infarction (OR 1.984, 95% CI 1.138–3.460) were independent risk factors for preoperative DVT in patients awaiting primary hip arthroplasty (Fig. 2).

### The risk factors of postoperative DVT

As shown in Table 3, the univariate analysis showed that there were significant differences in age ≥ 65 years (*P* < 0.001), hypertension (*P* = 0.022), tumor (*P* = 0.001), triglyceride (*P* = 0.032), preoperative *D*-dimer (*P* < 0.001), postoperative platelet (*P* = 0.009), and postoperative PT (*P* = 0.030) in DVT group compared with the non-DVT group.

**Table 3:** Univariate analysis of risk factors for postoperative DVT

Risk factors	DVT group (*n* = 38)	Non-DVT group (*n* = 530)	*P* Value
Age (≥ 65 years vs < 65 years)	36/2	300/230	< 0.001
Sex (women vs men)	28/10	306/224	0.054
BMI (≥ 25 vs < 25)	12/26	205/325	0.384
Hypertension (yes vs no)	22/16	207/323	0.022
Diabetes (yes vs no)	5/33	56/474	0.618
Cardiovascular disease (yes vs no)	6/32	62/468	0.453
Cerebral infarction (yes vs no)	9/29	79/451	0.149
Tumor (yes vs no)	6/32	20/510	0.001
Anticoagulation (yes vs no)	3/35	39/491	0.903
Triglyceride (mmol/L)	1.0(0.7, 1.4)	1.1(0.8, 1.5)	0.032
Cholesterol (mmol/L)	4.3 ± 1.0	4.8 ± 10.2	0.772
Apolipoprotein A (g/L)	1.1(0.9, 1.2)	1.0(0.9, 1.1)	0.156
Apolipoprotein B (g/L)	0.7(0.6, 0.9)	0.8(0.7, 0.9)	0.413
Preoperative hemoglobin (g/L)	129.0(117.3, 137.3)	128.0(117.0, 137.0)	0.911
Preoperative RBC (10^12/L)	4.2(3.9, 4.5)	4.2(3.9, 4.6)	0.760
Preoperative HCT (L/L)	37.6 ± 4.2	39.4 ± 10.0	0.268
Preoperative platelet (10^9/L)	196.0(155.5, 241.5)	190.0(155.0, 236.0)	0.567
Preoperative APTT (sec)	27.6(26.1, 28.3)	27.3(25.7, 29.1)	0.557
Preoperative PT (sec)	11.6(11.0, 12.1)	11.3(10.8, 12.0)	0.152
Preoperative TT (sec)	17.4(16.6, 18.8)	17.6(16.9, 18.5)	0.749
Preoperative fibrinogen (g/L)	3.3(2.7, 3.9)	3.0(2.6, 3.8)	0.504
Preoperative *D*-dimer (mg/L)	2.0(1.1, 6.7)	0.8(0.4, 1.9)	< 0.001
Duration of surgery (≥ 90 min vs < 90 min)	24/14	353/177	0.664
Intraoperative bleeding (ml)	150.0(100.0, 300.0)	200.0(100.0,250.0)	0.332
Postoperative bedrest time (day)	2.0(1.0, 3.0)	2.0(1.0, 2.0)	0.129
Postoperative drainage volume (ml)	140.0(50.0, 240.0)	120.0(60.0, 210.0)	0.718
Postoperative hemoglobin (g/L)	101.5(93.0, 111.3)	105.0(94.0,116.0)	0.278
Postoperative RBC (10^12/L)	3.4(3.1, 3.6)	3.4(3.1, 3.8)	0.345
Postoperative HCT (L/L)	30.7 ± 4.6	31.1 ± 5.0	0.682
Postoperative platelet (10^9/L)	202.0(182.0, 240.0)	178.0(142.0, 225.0)	0.009
Postoperative PT (sec)	12.3(11.4, 12.7)	11.6(10.9, 12.3)	0.030
Postoperative APTT (sec)	28.8(27.2, 32.7)	28.7(26.2,32.3)	0.465
Postoperative fibrinogen (g/L)	5.3 ± 1.1	5.1 ± 1.1	0.421
Postoperative TT (sec)	16.0(15.6, 16.5)	16.1(15.6, 16.7)	0.306
Postoperative *D*-dimer (mg/L)	2.2(1.5, 3.3)	1.9(1.3, 2.6)	0.109

*BMI* Body mass index, *RBC* Red blood cell, *HCT* Hematocrit, *APTT* Activated partial thromboplastin time, *PT* Prothrombin time, *TT* Thrombin time

**Fig. 3:**
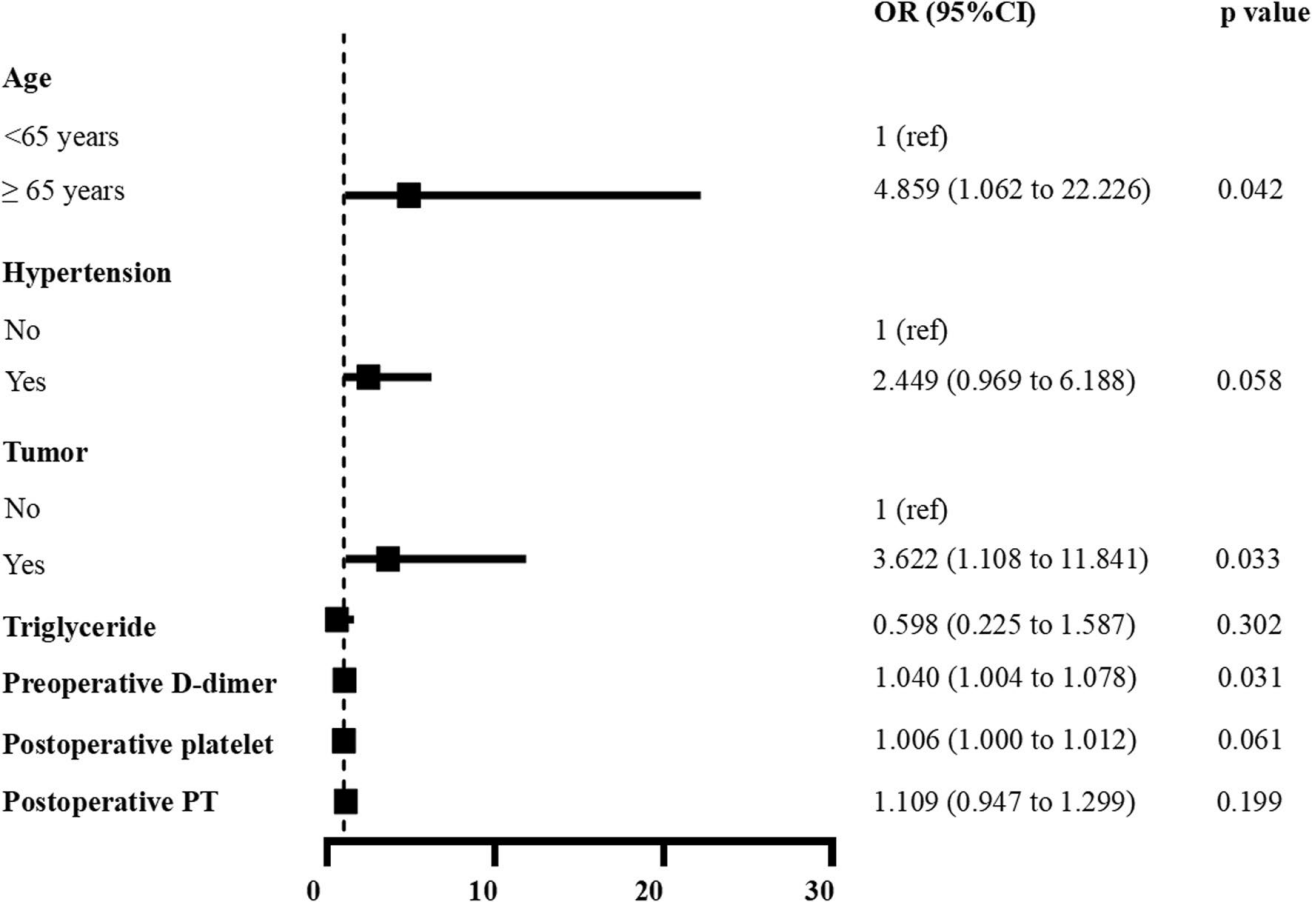
Multivariate analysis of postoperative DVT in patients undergoing unilateral primary hip arthroplasty via DAA

**Fig. 4:**
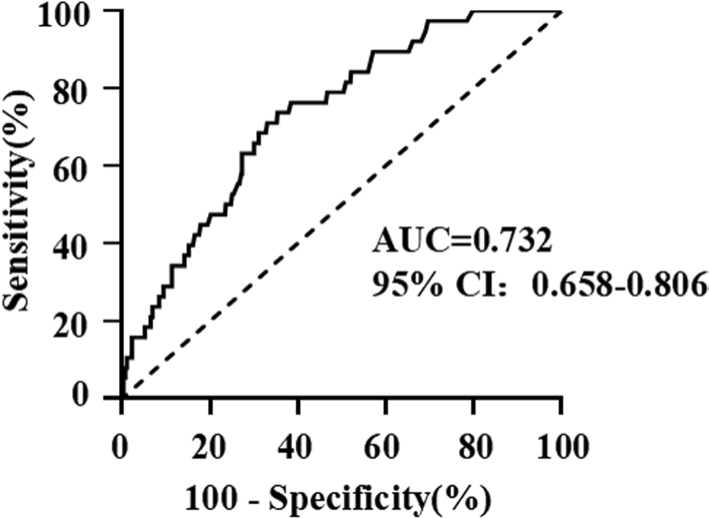
The ROC curve of preoperative *D*-dimer to assess the diagnostic value of postoperative DVT in patients undergoing unilateral primary hip arthroplasty via DAA

The subsequent multivariate logistic regression analysis showed that age ≥ 65 years (OR 4.859, 95% CI 1.062–22.226), tumor (OR 3.622, 95% CI 1.108–11.841), and preoperative *D*-dimer (OR 1.040, 95% CI 1.004–1.078) were independent risk factors for postoperative DVT (Fig. 3). ROC curve analysis showed that the best cutoff value of preoperative *D*-dimer in the diagnosis of new-onset DVT was 1.44 mg/L. The sensitivity was 73.7%, specificity was 64.6%, and the area under the curve was 0.732 (95% CI 0.658–0.806), *P* < 0.001, statistically significant (Fig. 4 and Table 4).

**Table 4:** Predictive value of preoperative *D*-dimer for postoperative DVT in patients undergoing unilateral primary hip arthroplasty via DAA

AUC	0.732
*P* value	< 0.001
Cutoff value (mg/L)	1.44
Sensitivity (%)	73.7
Specificity (%)	64.6

*AUC* Area under the curve

## Discussion

In the present study, most of the preoperative thrombus and newly formed postoperative thrombus were distal DVT of the affected side. The incidence of DVT before hip arthroplasty was 12.7% (83/651), which is in good accordance with those of previous reports (5.2–12.3%) [17–19]. Interestingly, the occurrence of new-onset postoperative DVT was 6.7% (38/568), which is lower than that reported by previous reports [20–22]. On the one hand, DAA may minimize the occurrence of femoral vein occlusion compared to other approaches, thus possibly reducing the incidence of postoperative DVT [23, 24]. On the other hand, the reported advantages of DAA include less soft tissue injury, less blood loss, fewer postoperative pain, faster recovery time, and shorter hospitalization compared with other traditional approaches, which have been reported to be associated with a lower incidence of DVT [25, 26].

Older age is a well-known risk factor for DVT in patients following surgery, which has been incorporated into some thrombosis prevention scoring systems [20, 27, 28]. Our results also showed that age ≥ 65 was the risk factor for both preoperative and postoperative DVT, which is in accordance with previous studies [8, 21, 29]. Possible reasons could be age-related remodeling of the venous wall [30, 31], low cardiorespiratory fitness, and diminished activity of the calf muscle pump in the elderly, all of which led to slow blood flow and high susceptibility to DVT.

In our research, we observed that female patients exhibited a higher incidence of both preoperative and postoperative DVT, which is consistent with previous studies [29, 32, 33]. The possible reason could be most of the subjects in our study were postmenopausal women and the change in sex hormone levels could cause dyslipidemia, which could increase the incidence of venous thromboembolism[34, 35]. Patients with cerebral infarction are more prone to venous thromboembolic events explained by longer bed rest time and a decrease in lower leg muscle pump action due to limb hemiplegia [36, 37]. Luanjiao Hu et al. found that the incidence of lower extremity DVT in patients with ischemic stroke hemiplegia is up to 18.57% and can drop to 1.89% after early rehabilitation nursing [38].

A previous study showed that the prevalence of venous thromboembolism in patients with malignant tumors was sevenfold higher than that of patients without malignant tumors [39]. In our study, 29 patients had a history of tumor, of whom 25 had a malignant tumor, one had a benign tumor, and 3 had a history of tumors without definite diagnosis. Our results also showed that patients who had a tumor history were more likely to develop postoperative DVT. On the one hand, tumor cells are known to secrete procoagulant active substances such as tissue factor, and this can cause hypercoagulability [40]. On the other hand, antitumor therapy may also increase the risk of DVT.

D-dimers are the products of fibrin degradation that appear in the blood after blood clot destruction and have been widely used for DVT screening [41–44]. However, *D*-dimer shows high sensitivity but low specificity because it may also be elevated in inflammatory, infection, trauma, surgery, bleeding, pregnancy, and cancer situations [43, 45]. This could be one possible explanation for the non-significant difference in *D*-dimer value between DVT and non-DVT groups on postoperative days 3 in this study. Thomas et al. [46] also showed that *D*-dimer is not a useful screening test for the diagnosis of DVT postoperatively. They determined that 92% of patients had serum *D*-dimer measurements higher than the institutional threshold (0.40 mg/ L) at 6 weeks after hip arthroplasty. Nevertheless, our results demonstrated that preoperative *D*-dimer is a potential predictor for postoperative DVT in patients undergoing DAA hip arthroplasty, and the optimal cutoff was 1.44 mg/ L by ROC curve. These findings were consistent with that reported by Yuichiro Shimoyama et al. [47] with difference in the preoperative plasma *D*-dimer cutoff value, which could be due to the difference in the study population and surgical approaches. Accordingly, if a patient’s preoperative *D*-dimer is above 1.44 mg/L, the clinicians should confirm whether the patient has symptoms such as pain or swelling of the affected limb and are encouraged to conduct an ultrasound examination of the lower extremities to identify postoperative DVT as early as possible.

This study has some limitations that warrant consideration. First, this study was a single-center retrospective study, thus limiting the external validity of our findings in other populations. Second, ultrasonography was performed only within postoperative 7 days, which is likely to ignore patients who developed DVT after discharge, thus underestimating the incidence of postoperative DVT. Last, as our study is limited due to its retrospective nature, other potential risk factors for DVT, such as preoperative activities of daily living impairment [48, 49], were not included in the analysis. It would be valuable to investigate the relationship between preoperative WOMAC score and the incidence of DVT. In the future, a prospective, multicenter, randomized controlled, large-sample study is needed to further explore the risk factors of perioperative DVT in patients requiring hip arthroplasty.

## Conclusions

In summary, the incidence of DVT in patients undergoing DAA hip arthroplasty was low, and the occurrence of DVT before and after unilateral primary hip arthroplasty performed through DAA was related to multiple factors. Clinicians and other medical staff should recognize these risk factors as early as possible to take effective prevention measures, thereby reducing the occurrence and development of perioperative DVT.

## Data Availability

All datasets used and analyzed during the current study are available from the corresponding author on reasonable request.
